# Physiotherapist-led ultrasound-guided visual biofeedback prelabor training: a randomised controlled trial

**DOI:** 10.1186/s12884-026-08976-8

**Published:** 2026-03-21

**Authors:** Noa Ben-Ami, Tamira Tischler, Sarit Reznik Ruff, Natav Hendin, Eran Hadar, Sofie Rousseau, Sharon Perlman

**Affiliations:** 1 Department of Physiotherapy, Ariel University, Ariel, Israel; 2 Gray School of Medicine, Faculty of Medical and Health Sciences, Tel Aviv University, Tel Aviv, Israel; 3 The Helen Schneider Women’s Hospital, Rabin Medical Center, Petach-Tikva, Israel; 4 The Paul Baerwald School of Social Work and Social Welfare, The Hebrew University, Jerusalem, Israel

**Keywords:** Visual biofeedback, Trans perineal ultrasound, Trans abdominal ultrasound, Pelvic floor muscle training, Prelabor education

## Abstract

**Background:**

Prenatal education improves maternal and neonatal outcomes. Ultrasound-guided visual biofeedback provides real-time visualization of fetal head movement, enabling expectant mothers to refine their pushing techniques. Pelvic floor muscle training may improve the ability to contract and relax the pelvic floor during labor. When used during the second stage of labor, US-BF has been shown to confer obstetrical benefits. Implementing prelabor ultrasound-guided visual biofeedback can further improve motor learning and optimize pushing effectiveness during the second stage of labor. This study aimed to evaluate the feasibility and effectiveness of pelvic floor physiotherapist-led prelabor ultrasound-guided visual biofeedback for improving obstetrical outcomes.

**Methods:**

A single-blinded randomized controlled trial was conducted in a community clinic. The study was approved by the Ariel University Human Ethics Committee (20211128) and registered on ClinicalTrials.gov (ID: NCT05258786). Nulliparous women at term with singleton pregnancies in vertex presentation were randomized into three groups: (1) Intervention: Prelabor transperineal and transabdominal ultrasound-guided visual biofeedback for pelvic floor muscle training and second-stage pushing training, (2) Control: Verbal instructions for pelvic floor muscle training and second-stage pushing, and (3) Routine Care: Standard prenatal education. In-person training sessions were held at an outpatient physiotherapy clinic between 37 and 39 weeks of gestation.

Within the intervention group, pushing efficacy was assessed using the angle of progression, and pelvic floor muscle contraction efficacy was assessed using bladder base displacement across the pre-, during-, and post-biofeedback phases. Obstetric outcomes, including second-stage duration, mode of delivery, perineal integrity, and postpartum urinary retention, were retrieved from medical records. Stress urinary incontinence was assessed using the International Consultation on Incontinence Questionnaire–Short Form before delivery and at two and eight weeks postpartum.

**Results:**

Out of 161 randomized women, 120 completed the study. In the intervention group, pushing efficacy showed a significant quadratic pattern, improving during biofeedback and declining after visual feedback was withdrawn (*p* = 0.007), while pelvic floor muscle contraction efficacy remained largely unchanged. Compared to the combined control and routine care groups, the intervention group had significantly lower rates of operative vaginal delivery (*p* = 0.050), higher rates of an intact perineum (*p* = 0.025), and lower rates of postpartum urinary retention requiring catheterization (*p* = 0.039). No significant differences were found in second-stage duration, cesarean delivery rates, or stress urinary incontinence outcomes.

**Conclusions:**

Physiotherapist-led prelabor ultrasound-guided visual biofeedback for second-stage pushing and pelvic floor muscle training was feasible and associated with improved obstetrical outcomes. These findings suggest a promising direction for enhancing prelabor education and maternal preparation.

The study was approved by the ethical review board (Ariel University Human Ethics Committee Noa Ben Ami − 20211128, Ariel University, Israel) and registered on ClinicalTrials.gov on February 18, 2022 (ID: NCT05258786). Prior to enrolment, informed consent was obtained from all participants. All procedures were conducted in accordance with the ethical standards of the institutional research committee, and the 1964 Declaration of Helsinki and its subsequent amendments.

**Supplementary Information:**

The online version contains supplementary material available at 10.1186/s12884-026-08976-8.

## Background

Prenatal education for preparing for childbirth has been shown to enhance maternal and neonatal outcomes and decrease childbirth anxiety [1, 2]. Among the advantages of introducing ultrasound into the delivery room is the ability to visually track the fetal head within the birth canal, enabling both delivery room staff and laboring women to observe fetal head movements in response to maternal pushing efforts. Intrapartum ultrasound-guided visual biofeedback (US-BF), administered by obstetricians during the second stage of labor, has been associated with improved pushing efficiency [3–5] However, inconsistent results have been reported regarding the effects on perineal integrity, mode of delivery, or the overall length of labor. In the psychological domain, US-BF during the second stage has been linked to enhanced maternal-newborn bonding and reduced postpartum stress [3, 6] Prelabor US-BF has shown promise in enhancing pushing effectiveness, increasing the likelihood of vaginal delivery, and reducing the duration of the first, second, and active second stages of labor [7].

Co-activation, or contraction of the levator ani during Valsalva, was reported to be associated with a prolonged second stage of labor [8, 9]. Maternal pushing associated with pelvic floor muscle relaxation is key to vaginal birth, and therefore, methods that improve control of pelvic floor function were mentioned as promising interventions [10].

Pelvic floor muscle training (PFMT) is a cornerstone in the customary management of stress urinary incontinence (SUI) [11–14]. Aimed at increasing muscle strength, endurance, and coordination, PFMT during pregnancy has been shown to be an effective preventive measure not only for reducing the risk of SUI but also for decreasing the incidence of third- or fourth-degree perineal tears. Transabdominal ultrasound has been shown to enhance PFMT and reduce the need for a digital patient examination [15, 16].

This study explores the synergistic effects of prelabor integrated US-BF for maternal training for second stage pushing and PFMT provided sequentially, led by a physiotherapist.

Nulliparous women who lack direct experience with their body’s functions during labor may benefit most from the sensorimotor information provided through visual biofeedback. We hypothesize that this dual intervention will improve maternal pushing efficacy and obstetrical outcomes.

## Methods

### Study design

A randomized, single-blinded, controlled trial was conducted in a community outpatient physiotherapy clinic. The study was approved by the institutional ethics committee (AU-HEA-NBA-20211128) and registered on ClinicalTrials.gov (NCT05258786). All participants provided written informed consent prior to enrollment.

The trial was designed with centralized, standardized training and decentralized, real-world obstetric care. All study sessions were delivered in a single outpatient physiotherapy clinic by the same trained physiotherapist using a standardized protocol, while participants were subsequently delivered in multiple hospitals according to routine obstetric care. Obstetric outcomes were retrieved from medical records by assessors blinded to group allocation.

### Participants and study groups

Nulliparous women with a singleton pregnancy in vertex presentation who planned a vaginal delivery were recruited through social media platforms. Exclusion criteria included multiple gestations, major fetal malformations, and severe fetal growth restriction. Participants were randomized in a 1:1:1 ratio to one of three groups using a pre-prepared allocation sequence with variable block sizes generated in advance. Participants were blinded to group allocation and to the specific study hypotheses. The physiotherapist delivering the intervention and the study coordinators were aware of group allocation, whereas obstetric outcome assessors and data analysts were blinded to group assignment.

Participants were allocated to one of three study groups: (1) the ultrasound-guided visual biofeedback (US-BF) intervention group, which received integrated transperineal and transabdominal US-BF for second-stage pushing training and pelvic floor muscle training (PFMT); (2) the control group, which received verbal instructions for second-stage pushing and PFMT without ultrasound guidance; and (3) the routine care group, which received standard prenatal care without any additional training sessions. Both the US-BF intervention and control groups attended a dedicated in-person session with the same physiotherapist and comparable time (45 min) devoted to instruction and interaction, in order to control for attention and therapeutic contact, with the only difference being the use of ultrasound-guided visual biofeedback in the US-BF intervention group.

The physiotherapist underwent structured training under the supervision of a senior obstetrician and a senior physiotherapist to ensure standardized delivery of the protocol. This training focused on the practical application of ultrasound-guided visual biofeedback for maternal training for second-stage pushing and pelvic floor muscle training.

In-person sessions were conducted between 37 and 39 weeks of gestation. Each participant in group (1) attended a single structured session lasting approximately 40–45 min, including explanation of the procedure, transabdominal pelvic floor muscle training with ultrasound visual biofeedback, and transperineal ultrasound-guided training for second-stage pushing. The session was structured into three predefined phases for each task: a pre-biofeedback phase without visual feedback, a during-biofeedback phase with real-time ultrasound visualization, and a post-biofeedback phase after visual feedback was withdrawn. Short rest intervals of approximately 30–60 s were provided between sets to limit fatigue. Participants in the control group attended a session of comparable duration and structure, receiving the same explanations and verbal guidance but without ultrasound visualization.

Ultrasound-guided visual biofeedback was performed with a portable ultrasound system equipped with a 6 MHz, 35-mm curved linear-array transabdominal transducer (Shanghai, China).

### Ultrasound-guided visual biofeedback for maternal training for the second stage pushing

After a verbal explanation, transperineal ultrasound examinations were performed with an empty bladder. The transducer was placed in the mid-sagittal plane, allowing visualization of the symphysis pubis and the fetal head. The angle of progression (AOP) was measured using the established reference line along the long axis of the symphysis pubis, according to the method described by Barbera and colleagues [17]. Participants in the US-BF intervention group were first instructed to push without visual feedback, then to repeat the maneuver while observing the fetal head descent on the screen after the relevant anatomical landmarks were explained, and finally to push again without viewing the screen, applying the US-BF experience. Three pushing efforts were performed at each phase. Images were recorded, and the AOP was measured offline by a senior obstetrician (S.P.), who was blinded to obstetric outcomes.

Participants in the US-BF group were not instructed to use a specific pushing technique. Instead, they were encouraged to push in the way they found most natural and comfortable, whether open-glottis or Valsalva pushing. This approach was chosen to avoid introducing unnecessary complexity or confusion in the instructions. If paradoxical co-activation was observed, participants were invited to try the alternative method. In such cases, additional targeted explanations were provided, as detailed in the intervention protocol (Supplementary File 1).

For transabdominal pelvic floor muscle training, participants in the US-BF group were examined in the supine position with a comfortably full bladder, achieved through standardized pre-session fluid intake and avoidance of voiding shortly before the session. The transducer was placed suprapubically to visualize the bladder base [12]. Participants were initially instructed to contract the pelvic floor muscles using the verbal cue “squeeze the anus” [16] without viewing the screen. The screen was then revealed, and the relevant anatomical landmarks (bladder and pelvic floor muscles) were explained, allowing participants to observe bladder base elevation during correct contraction. If no elevation was observed, additional explanations were provided. Participants then repeated the contractions without visual feedback, applying the US-BF experience. Three contractions were performed at each phase. Pelvic floor muscle contraction efficacy was assessed offline based on bladder base displacement, with an “upward contraction” defined as cranial displacement of the bladder base, indicating appropriate activation rather than paradoxical descent or no movement.

### Outcome measures

Measured outcomes for the intervention included pushing efficacy, defined as the difference between the AOP at rest and during maximal push, and pelvic floor muscle contraction efficacy, defined by the presence of upward bladder base displacement during voluntary contraction. Obstetric outcomes, including second-stage duration, mode of delivery, perineal integrity, and postpartum urinary retention (PPUR) requiring bladder catheterization, were retrieved from medical records. Midwives and obstetricians attending the deliveries were blinded to the biofeedback and questionnaire results. Stress urinary incontinence was assessed using the International Consultation on Incontinence Questionnaire–Short Form (ICIQ-SF) before delivery, within two weeks postpartum, and at eight weeks postpartum.

### Statistical analysis

An a priori power analysis was performed using G*Power version 3.1.9.4. [18]. The analysis indicated that for the most complex statistical model, a minimum sample size of 99 participants (at least 33 per group) was required, assuming a medium effect size, a power of 0.90, and an alpha level of 0.05. Based on previous experience with similar studies, a dropout rate of approximately 35% was anticipated. To account for potential attrition and to ensure adequate final power, oversampling was performed and at least 51 participants per group were recruited.

Statistical analyses were conducted using SPSS version 29 (IBM Corp., Armonk, NY, USA). Within group (1), repeated-measures analyses were used to assess short-term mechanistic effects of the biofeedback intervention by comparing AOP measurements and derived pushing efficacy across the three predefined phases: pre-biofeedback, during-biofeedback, and post-biofeedback. Linear and quadratic trends were examined to characterize changes across these phases. Pelvic floor muscle contraction efficacy, defined by the presence of upward bladder base displacement, was analyzed in a similar repeated-measures framework.

Between-group analyses were performed to compare obstetric outcomes among groups (1), (2), and (3). Continuous variables were compared using analysis of variance (ANOVA) or Student’s t-test, as appropriate, and categorical variables were compared using chi-square tests. For clinically relevant outcomes, follow-up pairwise comparisons were conducted between group (1) and each comparator group.

All statistical tests were hypothesis-driven and based on a priori directional hypotheses derived from previous intrapartum and prelabor ultrasound-guided biofeedback studies demonstrating improved pushing biomechanics and/or clinical outcomes. Accordingly, one-tailed p-values were used for these comparisons, and a significance threshold of *p* ≤ 0.05 was applied.

## Results

**Fig. 1:**
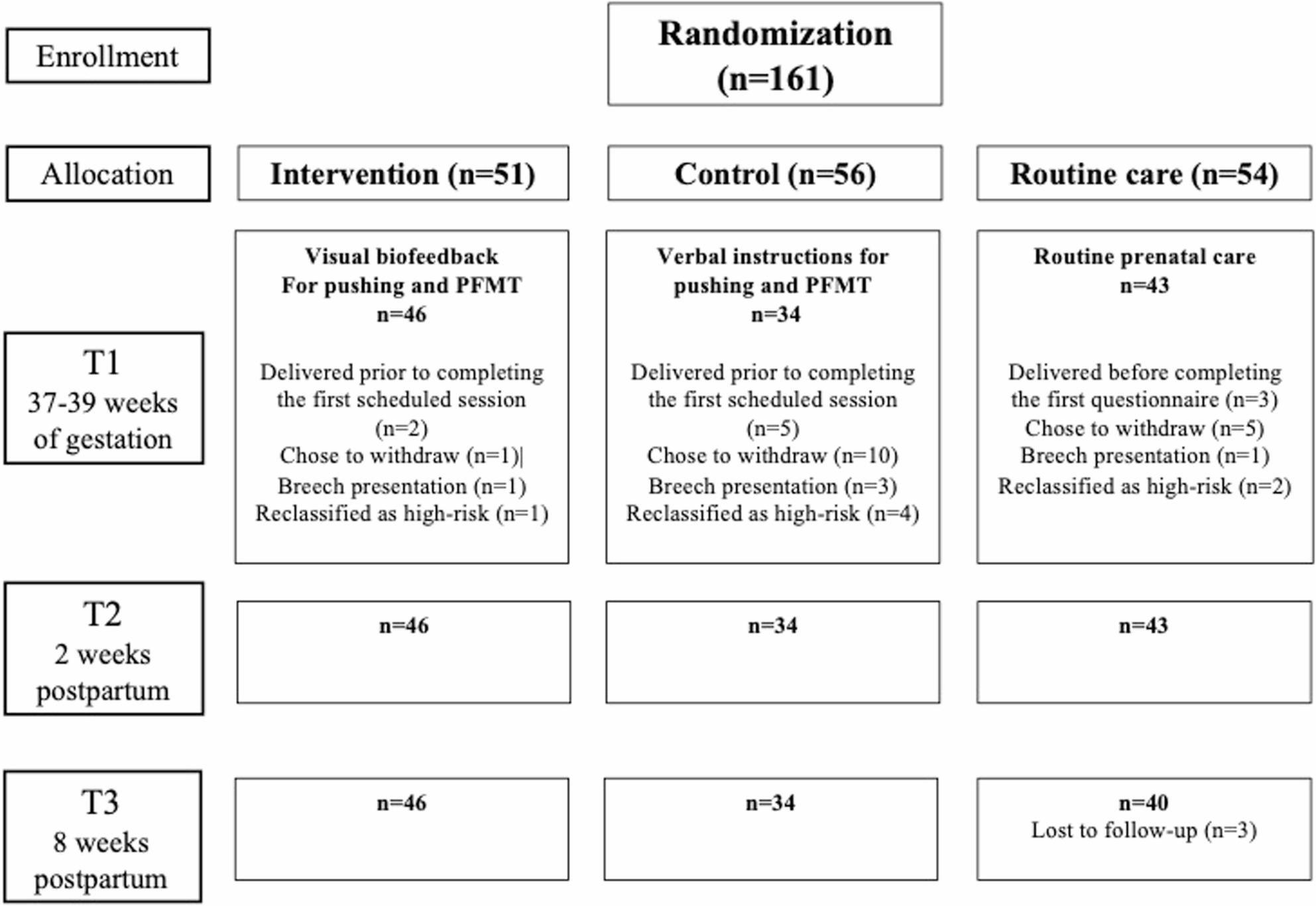
Study Design

A total of 161 participants were randomized, with 120 completing the study (Fig. 1). Several participants withdrew or were reclassified as high-risk pregnancies and were excluded before T1, and three participants from the routine care group were lost to follow-up at T3. For the analysis of obstetric outcomes and stress urinary incontinence at two weeks postpartum (T2), the intervention group comprised 46 participants, the control group 34 participants, and the routine care group 43 participants.

No statistically significant differences were found between the intervention, control, and routine care groups for maternal age, pregestational BMI, gestational age at delivery, labor induction, fetal sex, epidural analgesia during labor, or neonatal birth weight (Table 1). In addition to participating in the study, most participants in the intervention, control, and routine care groups attended public healthcare-led prenatal education classes, which primarily consisted of lectures (87%, 97.1%, and 86%, respectively; χ²(2) = 2.91, *p* = 0.11).

**Table 1:** Maternal and neonatal baseline demographic characteristics categorized by study group

	Intervention (*N* = 46)	Control (*N* = 34)	Routine care (*N* = 43)	Difference statistics
	Mean±SD	Mean±SD	Mean±SD	
Maternal age at recruitment (years)	31.62±3.77	30.15±4.05	31.14±3.8	F(2, 120) = 1.429, *p* = 0.122
Neonatal birth weight (kg)	3.18±0.34	3.17±0.29	3.26±0.36	F(2, 120) = 0.853, *p* = 0.215
Pregestational BMI	21.98±3.58	21.94±3.38	22.27±3.49	F(2, 97) = 0.079, *p* = 0.462
Gestational age at delivery	39.67±0.85	39.74±1.09	39.53±1.02	F(2, 98) = 0.361, *p* = 0.349
	**%**	**%**	**%**	
Gender infant female	47.8	52.9	39.5	Χ^2^(4) = 6.10, *p* = 0.096
Labor induction	45.7	44.1	41.9	Χ^2^(4) = 4.91, *p* = 0.149
Epidural analgesia	73.9	67.6	60.5	Χ^2^(4) = 4.77, *p* = 0.156
Attended prenatal class	87.0	97.1	86.0	Χ^2^(4) = 2.91, *p* = 0.117

### Efficacy of the ultrasound-guided biofeedback intervention

Within-participant repeated-measures analyses showed that across pre-, during-, and post-intervention measurement points, pushing efficacy did not change in a significant linear pattern (F(1,44) = 0.167; *p* = 0.685) but demonstrated a significant quadratic pattern (F(1,44) = 6.593; *p* = 0.007) (Table 2). Post hoc analyses indicated a significant increase between pre- and during-intervention measurements (F(1,44) = 6.094; *p* = 0.009), followed by a significant decrease between during-intervention and post-intervention measurements (F(1,44) = 4.299; *p* = 0.022). There was no significant difference between pre- and post-intervention measurements (F(1,44) = 1.262; *p* = 0.134).

**Table 2:** Intervention efficacy

	Mean ± SD	*P*-value
Baseline AoP		0.007^a^
Pre biofeedback	108.45°±14.64°	
biofeedback	109.78°±15.44	
Post biofeedback	109.94°±15.44	
Push AoP		
Pre biofeedback	121.56°±18.33°	
biofeedback	126.21°±20.31°	
Post biofeedback	123.54°±18.31°	
Push efficacy (ΔAoP)		
Pre biofeedback	12.97°±8.06°	
Biofeedback	16.45°±9.97°	
Post biofeedback	13.51°±8.69°	
	%	
Pelvic floor contraction (‘upward’)		0.311
Pre biofeedback	73.91%	
Biofeedback	76.09%	
Post biofeedback	78.26%	

^a^ Quadric changes

### Pelvic floor contraction efficacy

An “upward” contraction was observed in 73.91% of participants before the biofeedback, 76.09% during the biofeedback intervention, and 78.26% after the biofeedback. These changes were not significant (test of linear change: F(1,45) = 0.246; *p* = 0.311; test of quadratic change: F(1,45) = 0.000; *p* = 0.500) (Table 2).

### Obstetrical outcomes

Obstetrical outcomes and between-group analyses are presented in Table 3.

**Table 3:** Obstetrical outcomes categorized by study group

	Intervention (*N* = 46)	Control (*N* = 34)	Routine care (*N* = 43)	*P* ^a^
Duration of labor stages				
Duration second stage labor (minutes)	121.35 ± 230.29	113.22 ± 96.4	67.05 ± 77.36	0.180
Mode of delivery				
Spontaneous vaginal delivery	35 (76.09%)	22 (64.71%)	26 (60.47%)	
Operative vaginal delivery	6 (13.04%)	10 (29.41%)	9 (20.93%)	
Cesarian delivery (all)	5 (10.87%)	2 (5.88%)	8 (18.60%)	0.364
Cesarian delivery (during first stage of labor)	5 (10.87%)	2 (5.88%)	4 (9.30%)	
Cesarian delivery (during second stage of labor)	0 (0%)	0 (0%)	4 (9.30%)	

	Intervention (*N* = 41)	Control (*N* = 32)	Routine care (*N* = 35)	P^a^
Vaginal delivery				
Operative vaginal delivery	6 (14.63%)	10 (31.12%)	9 (25.71%)	0.050^b^
Perineal tearing	30 (73.17%)	30 (93.75%)	29 (82.86%)	0.025^b^
OASIS	1 (2.5%)	1 (3.23%)	3 (8.57%)	0.201
Post-partum urinary retention	4 (9.76%)	9 (28.13%)	7 (18.92%)	0.039^b^

Data are given as n(%) or mean ± SD

Statistical analysis was conducted to compare the intervention group with the control and routine care groups

^a^Calculated using Student’s t-test or chi-square test as appropriate, ^b^Statistically significant (*p* < 0.05)

No significant differences were observed between the intervention group and the combined control/routine care groups for the duration of the second stage of labor (*p* = 0.180).

Among participants who delivered vaginally, significantly fewer operative vaginal deliveries (vacuum-assisted or forceps-assisted) were observed in the intervention group compared with the combined control/routine care groups (14.63% vs. 28.36%, respectively; χ²(1) = 2.693; *p* = 0.050). Follow-up analyses showed that the rate of operative vaginal delivery in the intervention group (14.6%) was significantly lower than in the control group (31.3%; χ²(1) = 2.899; *p* = 0.044), whereas the difference between the intervention group (14.6%) and the routine care group (25.7%) did not reach statistical significance (χ²(1) = 1.463; *p* = 0.113). No significant differences were observed between the intervention group and the combined control/routine care groups in the incidence of cesarean delivery (10.87% vs. 12.98%, respectively; χ²(1) = 0.121; *p* = 0.364).

Significant differences were observed in the incidence of intact perineum between the intervention group and the combined control/routine care groups (26.83% vs. 11.94%, respectively; χ²(1) = 3.889; *p* = 0.025). An intact perineum was significantly more frequent in the intervention group (26.8%) than in the control group (6.3%; χ²(1) = 5.200; *p* = 0.012). The difference between the intervention group (26.8%) and the routine care group (17.1%) did not reach statistical significance (χ²(1) = 1.020; *p* = 0.156).

No significant differences were observed in the prevalence of grade 3–4 perineal tears between the intervention group and the combined control/routine care groups (2.5% vs. 5.97%, respectively; χ²(1) = 0.703; *p* = 0.201). These events were infrequent across groups.

Significant differences in the incidence of postpartum urinary retention requiring bladder catheterization during the postpartum hospitalization period were observed between the intervention group and the combined control/routine care groups (9.76% vs. 23.19%, respectively; χ²(1) = 3.119; *p* = 0.039). The incidence of postpartum urinary retention in the intervention group (9.76%) was significantly lower than in the control group (28.13%; χ²(1) = 4.143; *p* = 0.021) but was not significantly different from that in the routine care group (18.92%; χ²(1) = 1.348; *p* = 0.123).

### Stress urinary incontinence

The baseline incidence of urine leakage at T1 was 34.78% in the intervention group, 41.18% in the control group, and 6.98% in the routine care group. No significant differences were found when comparing urine leakage incidence between T1 and T2 (F(1,118) = 0.115; *p* = 0.368), T1 and T3 (F(1,118) = 0.002; *p* = 0.475), or T2 and T3 (F(1,118) = 0.102; *p* = 0.375). In addition, participation in the intervention group did not significantly affect changes in urinary incontinence questionnaire scores between T1 and T2 (F(1,118) = 0.052; *p* = 0.410), T1 and T3 (F(1,118) = 0.027; *p* = 0.435), or T2 and T3 (F(1,118) = 0.210; *p* = 0.324) (Table 4).

**Table 4:** Urinary incontinence analysis

	Intervention (*N* = 46)		Control (*N* = 34)		Routine care (*N* = 43)	
	M	SD	M	SD	M	SD
T1 urinary leakage questionnaire	2.35	3.62	2.85	3.77	0.51	2.27
T2 urinary leakage questionnaire	2.00	4.09	1.85	3.78	1.10	3.10
T3 urinary leakage questionnaire	2.07	4.22	2.21	4.01	0.35	1.55
	%		%		%	
T1 urinary leakage	34.78		41.18		6.98	
T2 urinary leakage	26.09		23.53		12.50	
T3 urinary leakage	26.09		26.47		5.00	

## Discussion

This study demonstrated that ultrasound-guided visual biofeedback (US-BF), using transperineal ultrasound for second-stage pushing training and transabdominal ultrasound for pelvic floor muscle training (PFMT), can be delivered by a physiotherapist in a standardized outpatient setting and is associated with improved obstetrical outcomes. To our knowledge, this is the first study to integrate prelabor training for second-stage pushing with PFMT in a single intervention session. Importantly, the focus of this work is not on provider proficiency, but on the feasibility and scalability of implementing this intervention within a multidisciplinary perinatal care model.

The improvement in pushing efficacy observed in our results aligns with previous studies conducted during the second stage of labor that involved brief interventions in the delivery room. ^3–5^ In our outpatient study, performed before labor onset, pushing efficacy showed a clear quadratic pattern: improvement during real-time biofeedback and a decline once visual feedback was withdrawn, with no significant difference between pre- and post-biofeedback within the same session. This pattern is consistent with findings from the motor-learning and biofeedback literature, in which performance typically peaks during concurrent feedback, whereas short-term retention depends on factors such as task complexity, practice amount, and fatigue. An immediate post-session assessment without feedback does not exclude meaningful learning or later retention. In pelvic floor rehabilitation and motor biofeedback research, visual biofeedback has been shown to enhance not only immediate performance but also subsequent execution and retention of correct movement patterns. Moreover, studies of obstetric ultrasound-guided biofeedback have demonstrated that improvements in biomechanics during coached pushing can translate into clinically meaningful outcomes even after brief training. In line with this, our clinical results showed fewer operative vaginal deliveries and better perineal outcomes compared with the verbal-instruction control group. These findings raise an important question for future studies, namely whether booster or repeated sessions closer to term could further enhance retention of the motor pattern and strengthen clinical effects, rather than indicating that learning did not occur.

Effective second-stage pushing requires relaxation of the pelvic floor muscles prior to the pushing effort to facilitate fetal head descent [8–10]. The obstetrical advantages observed in our study suggest that the integrated training for second-stage pushing and PFMT may have had a synergistic effect, enhancing the patient’s sense of control over pelvic floor muscle function. This likely resulted in more effective pushing and, consequently, a reduced incidence of operative deliveries, perineal trauma, and postpartum urinary retention (PPUR), all of which are associated with prolonged and ineffective pushing [19].

The duration of the second stage of labor was not shortened following the intervention, consistent with some previous reports [3, 5], although other studies have described a shorter duration [4, 8]. This discrepancy may reflect differences in local protocols, such as delayed pushing policies, as well as organizational factors including the ratio of women in labor to attending midwives, which vary across healthcare systems and countries.

Within the subset of women who delivered vaginally, those in the intervention group had significantly fewer operative vaginal births, consistent with a prior study using prelabor US-BF ^7^. As this effect was not consistently demonstrated when the intervention was performed intrapartum, it may suggest that prelabor training allows better assimilation of the motor pattern in a less stressful environment than the delivery room. This interpretation remains speculative and warrants further investigation.

An intact perineum substantially improves the overall labor and delivery experience, being associated with reduced pain, faster recovery, and fewer postpartum interventions. In our study, the incidence of intact perineum was higher in the intervention group than in the verbal-instruction control group, although the comparison with the routine care group did not reach statistical significance. Perineal integrity is a multifactorial outcome influenced by numerous maternal, fetal, and obstetric management factors, including fetal head size and position, duration of the second stage, use of operative vaginal delivery, episiotomy policy, perineal support techniques, maternal position, and epidural analgesia. Many of these variables cannot be fully standardized, particularly in a study in which women deliver in multiple institutions with different clinical protocols. The existing literature on ultrasound-guided biofeedback and perineal outcomes is also mixed [3–5], with some studies demonstrating reductions in perineal trauma and others showing improvements in pushing mechanics without a clear effect on tearing. This variability underscores that perineal trauma is not determined by pushing technique alone and that even mechanistically sound interventions may yield heterogeneous effects depending on context and co-interventions.

In this trial, the intervention group showed a significantly higher rate of intact perineum than the verbal-instruction control group, whereas the comparison with routine care did not reach statistical significance. Although the a priori power analysis indicated adequate power for the planned sample size, power calculations are necessarily based on assumptions about effect sizes and event rates that may not fully reflect the observed distribution of outcomes. This consideration is particularly relevant for binary outcomes, which are highly dependent on the number of observed events. Nevertheless, the observed differences were consistent in direction and magnitude with the hypothesized effect, and several comparisons between the intervention and routine care groups approached statistical significance. Notably, when the control and routine care groups were considered together, differences reached statistical significance, suggesting that limited power in subgroup comparisons may have contributed to the lack of statistical significance in some contrasts.

With regard to obstetric anal sphincter injuries (OASIS), events were infrequent in this cohort. Although the numerical difference between groups was clinically meaningful, the study was not specifically powered to detect differences in such low-incidence outcomes. The direction of effect, however, is consistent with the proposed mechanism of improved pelvic floor coordination and a reduced incidence of OASIS following PFMT [13].

The US-BF group exhibited a significantly reduced need for bladder catheterization for PPUR after vaginal delivery. In most cases, PPUR resolves early, but voiding difficulties and long-term urogenital morbidity may persist more often than previously thought, and for these patients, the consequences are devastating [20, 21]. To our knowledge, no previous studies have examined the effect of ultrasound-guided biofeedback on this outcome. Given the multifactorial and incompletely understood pathophysiology of PPUR, it is plausible that improved coordination between abdominal pushing and pelvic floor muscle relaxation reduced periurethral edema and pudendal nerve compression, thereby lowering the risk of transient bladder dysfunction.

Although extensive reviews support the effectiveness of PFMT in reducing stress urinary incontinence (SUI) [22–24], in the present study, the US-BF intervention did not significantly affect the prevalence of urine leakage or incontinence scores at any of the assessed time points. The discrepancy with the literature may relate to the reliance on self-reported measures, cultural factorssocial stigmas [25], or normalization of urinary incontinence as pregnancy progresses, leading to underestimations of symptom severity, as suggested by [26]. and the postpartum period. In addition, our intervention consisted of a single session, whereas most studies demonstrating a reduction in SUI involve repeated and sustained PFMT programs.

Several limitations should be acknowledged. First, unlike previous single-center studies of US-BF, participants in our study gave birth in multiple delivery wards with differing clinical protocols. This design enhances external validity and generalizability but also introduces potential inter-ward variability that could not be fully controlled. Second, recruitment through social media may have introduced selection bias. Third, most participants attended public prenatal education classes that included verbal instructions on PFMT and second-stage pushing, as well as recommendations for regular exercise and perineal massage, and individual adherence to these recommendations could not be controlled.

In addition, the study included a single prelabor training session and a relatively short 8-week postpartum follow-up period, which may not fully capture the persistence of learned motor patterns or longer-term pelvic floor outcomes.

Finally, the comparison between the verbal-instruction control group and the routine care group merits consideration. The control group represents a partial intervention without objective performance feedback, whereas routine care involves no additional training. In behavioral and physiotherapy research, such partial interventions can sometimes have unintended effects, as participants may attempt to modify behavior without sufficient feedback to guide execution, potentially leading to maladaptive strategies. This may partly explain why the control group occasionally performed worse than the routine care group. The use of an active control remains a methodological strength, as it helps distinguish the specific effect of biofeedback from the effects of attention and education alone, but the relatively modest sample size of this pioneering study may have limited the precision of some subgroup comparisons.

The statistical analyses were based on directional, hypothesis-driven expectations from previous ultrasound-guided biofeedback studies. Therefore, one-tailed testing was employed, and no overall correction for multiple comparisons was applied, as such corrections can greatly increase the risk of type II errors in studies with limited sample sizes and are not ideal for conceptually distinct, predefined hypotheses. Larger future studies will permit more rigorous statistical methods while still maintaining sufficient power.

## Conclusions

This randomized controlled trial demonstrated that a physiotherapist-led, prelabor ultrasound-guided visual biofeedback intervention integrating second-stage pushing and pelvic floor muscle training is feasible and is associated with improved obstetrical outcomes, including lower rates of operative vaginal delivery, perineal trauma, and postpartum urinary retention.

Although the study groups were relatively small, this work is pioneering in introducing an integrated biofeedback approach delivered by a physiotherapist rather than an obstetrician and in demonstrating both obstetrical benefits and potential improvements in postpartum recovery.

These findings provide a strong basis for larger follow-up studies to verify the observed effects and to further investigate differences between the intervention and standard care groups. This approach supports patient-centered care by enhancing maternal engagement in the childbirth process and is a meaningful innovation in prelabor education. Future research should explore its applicability in various professional settings, the best timing and frequency of training, the inclusion of women with diverse obstetric histories, and longer-term maternal outcomes.

## Supplementary Information

Supplementary Material 1.

Supplementary Material 2.

## Data Availability

The datasets used and/or analysed during the current study are available from the corresponding author on reasonable request.
