# Supplementary material for: Physiotherapist-led ultrasound-guided visual biofeedback prelabor training: a randomised controlled trial
Source: BMC Pregnancy Childbirth. 2026 Mar 21;26:471. doi: 10.1186/s12884-026-08976-8 (PMC13127039; doi:10.1186/s12884-026-08976-8)
Supplement: Supplementary file 1 — Supplementary Material 1. [file 12884_2026_8976_MOESM1_ESM.docx]

Intervention Group Protocol (Education and Ultrasound Biofeedback)

**Introduction and Education**

The session was conducted individually by a pelvic floor physiotherapist (participant seated at a table).

The objectives of the session were:

1. To introduce the anatomy and function of the female pelvic floor.
2. To briefly explain the stages of labor.
3. To teach voluntary pelvic floor muscle (PFM) contraction and relaxation.
4. To teach pushing techniques for the second stage of labor.

Using a female pelvic model, the physiotherapist explained that the pelvic floor forms the inferior part of the pelvis and consists of voluntary muscles under conscious control. These muscles can be actively contracted and relaxed, although they also function reflexively.

The bladder lies anteriorly, the uterus superiorly, and the rectum posteriorly. Externally, three openings are visible: the anus, the vagina (through which the baby is delivered), and the urethra (through which urine exits). The pelvic floor muscles encircle these three openings. During contraction, the openings close and lift upward and inward; during relaxation, they descend and open.

Participants were informed that during pelvic floor contraction, the bladder base elevates, which can be visualized on ultrasound as upward movement of the bladder (appearing as a hypoechoic/black structure). During increased intra-abdominal pressure (e.g., coughing, sneezing, laughing, lifting), the pelvic floor descends, and the bladder base moves downward. Failure to contract the pelvic floor prior to increased pressure may result in urinary leakage.

Participants were told that they would learn to voluntarily contract and relax the pelvic floor muscles, including anticipatory contraction (“the Knack”) to prevent stress urinary leakage during pregnancy and postpartum. It was also explained that during the second stage of labor, relaxation of the pelvic floor muscles is important to facilitate fetal head descent.

**Brief Overview of the Stages of Labor**

The first stage begins with the onset of regular contractions (occurring every 3–5 minutes) and ends with full cervical dilation.
The second stage begins with full dilation and ends with delivery of the baby.
The third stage begins with delivery of the baby and ends with delivery of the placenta, typically lasting 5–30 minutes.

Using a second pelvic model, fetal head descent through the pelvis and stretching of the perineum were demonstrated, including explanation of perineal tearing.

Participants were asked to confirm that they had consumed at least 600 mL of water prior to the session.

**Part 1: Pelvic Floor Muscle Training and Bladder Ultrasound Biofeedback**

The participant lay supine, clothed, on a disinfected examination bed with a pillow under the head and knees.

**Teaching the “Knack”**

Participants were first asked to cough forcefully and notice pelvic floor descent. They were then instructed to perform a maximal, rapid contraction of the anal sphincter immediately before coughing and to maintain the contraction during the cough.

Participants were asked whether they perceived a difference between coughing with and without anticipatory contraction.

**Transabdominal Ultrasound Biofeedback**

Participants were informed that the examination was external. The transducer was disinfected, gel applied, and placed suprapubically over the lower abdomen.

Participants first performed three maximal voluntary contractions without visual feedback.
For each contraction:

- One ultrasound image was saved at rest.
- One image was saved at maximal contraction.

A total of three images at rest and three at maximal contraction were stored.

The screen was then turned toward the participant. The bladder was identified on the screen, and participants were shown bladder base elevation during contraction and descent during relaxation.

Participants were asked to cough while observing the screen to visualize bladder descent. They were then instructed to perform the Knack (maximal contraction prior to coughing) and maintain contraction during coughing to prevent bladder descent.

Three additional contractions were performed without screen visualization, with images saved as described above.

For each saved contraction, the physiotherapist documented whether:

- Contraction was performed correctly (cranial bladder lift),
- No contraction was observed,
- Downward displacement (bearing down) occurred.

**Home Exercise Instructions**

Participants were instructed to perform pelvic floor muscle training three times daily:

- 10 short contractions (1-second contraction/relaxation),
- 10 sustained contractions (10 seconds each),
  with 20 seconds rest between sustained contractions,
  five days per week for six months.

They were instructed to perform anticipatory contraction before physical effort and relax afterward.

Participants were then asked to empty their bladder before proceeding.

**Part 2: Ultrasound Biofeedback for Second-Stage Pushing**

Bladder emptiness was confirmed via transabdominal ultrasound.

Participants were given privacy to remove underwear and were draped with a clean sheet.

**Transperineal Ultrasound Assessment**

Participants were informed that this was an external examination.
The transducer was disinfected, covered with a glove and gel, and placed on the perineum. Correct visualization of the fetal head (and not another presenting part) was confirmed.

**Pushing Without Visual Feedback**

Participants were instructed to push as if delivering the baby, without viewing the screen. Three pushes were performed.

For each push:

- One image was saved at rest (fetal head position at baseline),
- One image was saved at maximal push.

A total of three rest images and three maximal push images were stored.

**Pushing With Visual Biofeedback**

The screen was turned toward the participant. The symphysis pubis and fetal head were identified. Participants were asked to cough to visualize fetal head movement.

They were then instructed to push and direct the fetal head along the birth canal trajectory (demonstrated visually as a curved path on the screen).

Three pushes were performed with visual feedback, with images saved as described above.

**Pushing Without Visual Feedback (Motor Recall)**

Participants then practiced three additional pushes without viewing the screen, while mentally recalling the correct direction and effort learned during biofeedback. Images were saved as previously described.

**Closing Instructions**

Participants were instructed to remember the sensation and direction of effective pushing for use during labor, particularly if epidural anesthesia reduced sensory feedback.

The session concluded with encouragement and reinforcement.
