# Supplementary material for: Physiotherapist-led ultrasound-guided visual biofeedback prelabor training: a randomised controlled trial
Source: BMC Pregnancy Childbirth. 2026 Mar 21;26:471. doi: 10.1186/s12884-026-08976-8 (PMC13127039; doi:10.1186/s12884-026-08976-8)
Supplement: Supplementary file 2 — Supplementary Material 2. [file 12884_2026_8976_MOESM2_ESM.docx]

Control Group Protocol (Education Without Ultrasound)

**Introduction and Education**

The session was conducted individually by a pelvic floor physiotherapist (participant seated at a table).

The objectives of the session were:

1. To introduce the anatomy and function of the female pelvic floor.
2. To teach voluntary pelvic floor muscle (PFM) contraction and relaxation.
3. To provide a brief explanation of the stages of labor.

Using a female pelvic model, the physiotherapist explained that the pelvic floor forms the inferior part of the pelvis and consists of voluntary muscles under conscious control. These muscles can be actively contracted and relaxed, although they also function reflexively.

The bladder lies anteriorly, the uterus superiorly, and the rectum posteriorly. Externally, three openings are visible: the anus, the vagina (through which the baby is delivered), and the urethra (through which urine exits). The pelvic floor muscles surround these three openings. During contraction, the openings close and move upward and inward; during relaxation, they descend and release.

Participants were informed that during pelvic floor contraction the bladder base elevates (which can be visualized on ultrasound, although ultrasound was not used in this group). During increased intra-abdominal pressure (e.g., coughing, sneezing, laughing, lifting a baby), the pelvic floor descends and the bladder moves downward. If the pelvic floor is not contracted prior to increased pressure, urinary leakage may occur.

Participants were told that they would learn to voluntarily contract and relax the pelvic floor muscles in order to help prevent urinary leakage during pregnancy and postpartum. It was also explained that during the second stage of labor, relaxation of the pelvic floor muscles is important to facilitate fetal head descent.

**Brief Overview of the Stages of Labor**

The first stage begins with the onset of regular contractions (occurring every 3–5 minutes) and ends with full cervical dilation.
The second stage begins with full dilation and ends with delivery of the baby.
The third stage begins with delivery of the baby and ends with delivery of the placenta, typically lasting 5–30 minutes.

Using a pelvic model, fetal head descent through the pelvis and stretching of the perineum were demonstrated, including a brief explanation of possible perineal tears.

**Pelvic Floor Muscle Training (Without Ultrasound Biofeedback)**

The participant lay supine, clothed, on a disinfected examination bed with a pillow under the head and knees.

**Breathing for Relaxation**

Participants were instructed to take a slow breath in, directing the air into the abdomen (abdominal breathing) without expanding the chest. This was repeated three times.

It was explained that this type of breathing promotes pelvic floor relaxation and is important during the second stage of labor.

**Sustained Contractions (Endurance Training)**

Participants were asked to contract the anal sphincter three times, hold the contraction for several seconds, and then relax.

The purpose of these contractions was explained as improving pelvic floor muscle endurance. After each contraction, participants were asked whether they felt the anus lift upward.

**Quick Contractions**

Participants then practiced three rapid contractions and relaxations of the anal sphincter.

**Teaching the “Knack”**

Participants were first asked to cough forcefully and notice what they felt in the pelvic floor (e.g., downward pressure).

They were then instructed to perform a maximal, rapid contraction of the anal sphincter immediately before coughing and to maintain the contraction during the cough. They were asked whether they perceived a difference between coughing with and without anticipatory contraction.

**Home Exercise Instructions**

Participants were instructed that if they experienced urinary leakage during pregnancy or postpartum, they should perform pelvic floor muscle training:

- Three times per day
- 10 short contractions (1-second contraction/relaxation)
- 10 sustained contractions (10 seconds each)
- 20 seconds rest between sustained contractions
- Five days per week
- For six months

They were instructed to perform anticipatory contraction (“the Knack”) before physical effort and relax afterward.

The session concluded with encouragement.
